# Effects of a Combination of Water-Soluble Coenzyme Q10 and Collagen on Skin Parameters and Condition: Results of a Randomised, Placebo-Controlled, Double-Blind Study

**DOI:** 10.3390/nu12030618

**Published:** 2020-02-27

**Authors:** Katja Žmitek, Janko Žmitek, Mirjam Rogl Butina, Tina Pogačnik

**Affiliations:** 1 Institute of Cosmetics, VIST—Higher School of Applied Sciences, Gerbičeva ulica 51A, SI-1000 Ljubljana, Slovenia; janko.zmitek@vist.si (J.Ž.); mirjam.rb@dermatologija.eu (M.R.B.); tina.pogacnik@vist.si (T.P.); 2 Nutrition Institute, Tržaška cesta 40, SI-1000 Ljubljana, Slovenia; 3 Dermatologija Rogl Fabjan, Derčeva ulica 35, SI-1000 Ljubljana, Slovenia

**Keywords:** Coenzyme Q10, Q10Vital, CoQ10, collagen peptides, skin health, dermis density

## Abstract

Skin is a complex and dynamic organ that provides a protective interface between the external environment and the body; changes in skin appearance are often the first visible signs of aging. It is well established that nutrients and other bioactive substances have important roles in the structure and functions of human skin; however, the effects of dietary supplementation of such bioactives are much less investigated. The objective of this randomised, double-blind placebo-controlled study was to investigate the effects of liquid food supplement, characterised by a combination of water-soluble coenzyme Q10 (Q10Vital®) and collagen, on dermal density and other skin parameters in comparison to placebo. The trial was performed on 34 healthy women aged 40–65 that received either the test product (*n* = 17) or the placebo (*n* = 17) for twelve weeks. Measurements and assessments of skin parameters were performed at baseline and after 12 weeks of intervention. We observed improved dermis density, reduced periorbital wrinkle area and the total wrinkle score, and improved skin smoothness. On the other hand, changes in skin hydration, dermis thickness, transepidermal water loss (TEWL) and viscoelasticity were not significant.

## 1. Introduction

Increased life expectancy and quality is one of the greatest global public health challenges. Today, most people can expect to live into their 60s and beyond, and by 2050, the proportion of the world’s population over 60 years old is expected to nearly double from that in 2015, reaching 22% [1]. The shift in distribution of a population towards older ages gives rise to age-related challenges and also increased interest in combating the visible signs of skin aging. People are more preoccupied with looking and “staying” young, and research into the process of aging has expanded [2]. Skin is a complex and dynamic organ that provides a protective interface between the external environment and the body. Changes in its appearance are often the first visible signs of aging and this can have implications for emotional and mental wellbeing [2].

Being in direct contact with the environment, the skin is constantly exposed to external stress factors, and therefore skin aging is a complex process affected by both genetic (intrinsic aging) and environmental factors, such as sun exposure and ultraviolet (UV) irradiation, smoking, pollution, sleep deprivation and nutritional factors, thus characterizing extrinsic aging [3,4]. Among all environmental factors, UV irradiation is considered to be the main factor responsible for premature skin aging, leading to photoaged skin [5]. 

The effects of intrinsic and extrinsic aging commonly overlap, leading to structural and functional changes of all skin layers, with the most prominent modifications being found in the dermis. In the dermis, the densities of collagen, elastin and other dermal extracellular matrix components decrease, leading to a loss of volume. The dermal collagen network becomes fragmented and less organized and the elastic fibres lose their integrity [6]. While the increased expression of matrix metalloproteinases (MMPs) results in accelerated degradation of collagen and other extracellular matrix components, their synthesis by dermal fibroblasts slows down and is therefore unable to adequately replace the degraded matrix [7,8]. Imbalance between degradation and synthesis of the dermal matrix components results in a loss of strength and elasticity, leading to increased laxity, sagging, wrinkling, and rough-textured appearance of the skin [3,9].

The formation of free radicals is a widely accepted pivotal mechanism leading to skin aging. Free radicals and other reactive oxygen species (ROS) are inevitable coproducts in the metabolic processes and their formation occurs also due to exposure to external factors, such as UV irradiation [10]. Although the skin possesses a complex antioxidant defense mechanism, its efficiency decreases with age and the quantity of free radicals increases. This imbalance leads to the progressive deterioration of cellular structures, resulting in accelerated aging and skin immune system dysregulation [11].

It is well established that nutritional status with respect to macronutrients, micronutrients, and other bioactive constituents is important for skin health and appearance, and a wide range of nutritional supplements have been reported to have beneficial effects on skin health and more youthful appearance [4,12,13]. However, the scientific evidence for skin health-related claims is often scarce and is often based on in vitro or uncontrolled in vivo studies. 

In our recent placebo-controlled trial, we investigated the effects of supplementation with coenzyme Q10 (CoQ10) on skin properties [14]. The results show several beneficial effects for wrinkle reduction and improvement in skin smoothness, while the influence on dermis density and thickness was insignificant. Additionally, several studies showed the positive effects of collagen [15,16,17] or a combination of collagen with various other nutrients on skin parameters [18,19,20,21,22,23]. On the other hand, to our knowledge, the influence of a combination of CoQ10 and collagen on dermal density has not yet been investigated in any placebo-controlled human intervention. 

The purpose of this randomised, double-blind placebo-controlled study was to investigate the effects of the liquid food supplement, characterised by a combination of water-soluble CoQ10 and fish collagen, on dermal density and other skin parameters in comparison to placebo.

## 2. Materials and Methods 

### 2.1. Study Design 

The study employed a randomised, placebo-controlled, double-blind single period design. It was in full compliance with the principles laid out in the Declaration of Helsinki. The study protocol was approved by the Ethics Committee of the Higher School of Applied Sciences (2018/4-ET-SK) and included in the ClinicalTrials.gov register under the record NCT03811756.

### 2.2. Study Participants

Thirty-four healthy Caucasian female subjects, ranging in age from 40 to 65 years (mean age 54.4 ± 6.8 (SD)) with Fitzpatrick skin phototypes II and III and with signs of skin aging (mimic wrinkles/poor skin tone/visual dryness) were enrolled in the study after providing written consent. The exclusion criteria included pregnancy or breastfeeding, a known or suspected allergy to any ingredient of the tested products, changes in dietary habits and dietary supplementation in the last three months prior to inclusion, regular use of food supplements containing CoQ10 or other antioxidants, collagen or other protein-based food supplements, vitamin A, vitamin C or biotin in the last three months prior to inclusion, veganism, changes in cosmetic facial and body care routine in the last month prior to inclusion, diagnosed and uncontrolled/untreated/unregulated disease, any clinically significant history of serious metabolic disease, digestive tract disease, liver disease, kidney disease, haematological disease, acute skin diseases, skin pigmentation disorders, increased cholesterol and use of cholesterol lowering drugs (statins), anticipated sunbathing or solarium visits before or during the study, microinvasive and invasive rejuvenation treatments (e.g., needle rollers, needle mesotherapy, deep/medium-deep chemical peels etc.) in the last 6 months prior to study entry, non-invasive rejuvenation treatments (e.g., radiofrequency, electrotherapy, ultrasound therapy) in the last 2 months prior to study entry, and mental incapacity that precludes adequate understanding or cooperation. Subjects were asked not to change their routinely used skin care regimen on the test sides during the entire study period and to continue their normal dietary habits. All subjects provided signed informed consent before recruitment. 

Subjects’ compliance with the inclusion and exclusion criteria was checked before their inclusion in the study. Forty subjects were assessed for eligibility and while 6 did not meet the inclusion criteria, 34 subjects were enrolled into the study. They were randomly assigned to either (a) a placebo group (mean age 55.0 ± 7.6 years) or (b) a test group (53.7 ± 6.2 years) receiving test syrup, with 17 subjects per group; there was no significant difference in age between groups (*p* = 0.59). Randomisation was performed using a simple randomisation procedure (computerized random numbers).

### 2.3. Study Products and Intervention

All subjects consumed 10 mL of a syrup daily for 12 weeks with a meal. The test group received the investigational product—the test syrup (active ingredients per 10 mL: hydrolysed fish collagen: 4000 mg, water-soluble CoQ10 (Q10Vital^®^, Valens Int. d.o.o., Šenčur, Slovenia): 50 mg, vitamin C: 80 mg, vitamin A: 920 µg, biotin: 150 µg) and the placebo group received 10 mL of flavoured and coloured placebo syrup without any active ingredients. To enable the production of aqueous syrup with CoQ10, a water-soluble form of CoQ10 (Q10Vital^®^) [24,25] with improved bioavailability [26] was used. Both syrups were formulated and produced by Valens Int. d.o.o. (Šenčur, Slovenia) following good manufacturing practice guidelines. The composition of both investigated syrups was examined by an independent accredited laboratory (Chelab S.r.l, Resana, Italy).

### 2.4. Assessments

Regular checks of the subjects were carried out three times during the study: at the baseline (T0), after 6 weeks (T6) and after 12 weeks of supplementation (T12). During the whole 12-week intervention period, subjects kept a diary of test product intake, which was checked after 6 and 12 weeks of intervention (T6 and T12). Measurements and assessments of skin parameters were performed at baseline and after 12 weeks of intervention (T0 and T12). The results were obtained during a period of colder outside temperatures and low sun exposure from October 2018 to February 2019 (average monthly temp of 13.2 °C, 8.2 °C, 2.2 °C, 0.7 °C, 4.9 °C, respectively). All measurements were carried out on subjects lying in a room with a temperature of 20–25 °C and relative humidity 40–60%, except the Visioface imaging was done in a sitting position. Dermis density and thickness were measured on the right zygomatic area (approx. 2 × 2 cm), viscoelasticity (VE) measurements were performed on a predetermined area of the right cheek (approx. 2 × 2 cm), hydration and TEWL measurements were performed on a predetermined area of the left cheek (approx. 2 × 2 cm each). Measurements started after a 30 min acclimatisation period in the same atmospheric conditions. Subjects were advised to clean their face at least 2 h before the time of measurement and to not apply any cosmetic products on their face 2 h or less before the measurement.

### 2.5. Dermis Density and Thickness Measurements 

Dermis density and thickness were measured using ultrasonography with a DermaLab Series, SkinLab Combo, 20 MHz ultrasound probe (Cortex Technology ApS, Hadsund, Denmark) on a predetermined position on the right zygomatic area at the baseline and after 12 weeks of intervention as published elsewhere [27]. A constant gain curve was applied for each volunteer and ultrasonographic images were visualized on-screen and recorded. The integrated software of DermaLab (SkinLab, Cortex Technology ApS, version 1.04.1, Hadsund, Denmark) was used for digital image analysis. The epidermis was characterized by a hyper-reflecting band; the sub-epidermal hypo- and the hyper-echogenic bands corresponding, respectively, to papillary and reticular dermis were selected on the screen and dermis thickness as well as density were calculated on the whole skin block. Measurements were repeated three times and the average was calculated. Dermis density is measured as intensity with a 0–100 score and dermis thickness is measured in µm.

### 2.6. Skin Viscoelasticity, Hydration and TEWL Measurements

Viscoelasticity (VE) measurements were performed on a predetermined position of the right cheek using a DermaLab Series, SkinLab Combo, elasticity probe (Cortex Technology ApS, Hadsund, Denmark) with settings for normal skin condition (400 mbar negative pressure) as published elsewhere [27] at the baseline and after 12 weeks of intervention. Measurements were repeated three times. The VE value is the ratio of elastic recovery to the total deformation and represents biological elasticity. The measurement gives results in MPa, and higher values correspond to better skin viscoelasticity.

Hydration measurements were performed on a predetermined area of the left cheek using a DermaLab Series, SkinLab Combo hydration probe (Cortex Technology ApS, Hadsund, Denmark), which operates on the conductivity principle. Eight consecutive measurements were conducted, and the average was calculated. The measurement gives results in µS.

Transepidermal water loss (TEWL) measurements were performed on a predetermined position of the left cheek using a DermaLab Series, SkinLab Combo, TEWL probe (Cortex Technology ApS, Hadsund, Denmark). The TEWL depends on the diffusion of water through the stratum corneum [28] and is measured in g/m^2^/h. 

### 2.7. Photography, Wrinkle Measurements and Evaluation, Skin Smoothness and Microrelief Evaluations

High resolution lateral (left and right) and frontal images of the face (10 Mpx) were taken using the VisioFace Quick system (Courage + Khazaka electronic GmbH, Köln, Germany), with a constant distance from the camera in a light facial booth. The software-controlled diodes illuminate the face evenly. As the topography of the skin varies significantly within a few millimetres, the exact location of the face was obtained by exactly matching the positioning of the face in the booth to the baseline picture. Right lateral images were used for subsequent analysis according to procedure described elsewhere [29]. Assessment area on each image was exactly defined for all images using predefined anatomical landmarks on the face. The assessment area for each image was analysed using VisioFace CSI software version 3.6.2.1 (Courage+Khazaka electronic GmbH, Köln, Germany) analysis logarithms that automatically identified and quantified wrinkles. The wrinkle area fraction (wrinkle area divided by the assessment area) of periorbital wrinkles was measured for each subject at the baseline and after 12 weeks of use. 

Wrinkle severity assessment was also performed according to the objective visual scoring system, the Lemperle Scale [30]. Five different wrinkle types in different face areas (horizontal forehead lines (HF), glabellar frown lines (GF), eye (PO, periorbital lines), nasolabial folds (NL), and corner of the mouth lines (CM)) were graded on a scale 0–5 (0-none, 5-severe) by three experienced professionals at baseline and after the 12-week intervention using frontal and lateral Visioface images. The average wrinkle score was calculated for individual areas and the summation of the scores from individual sites provided the “Total wrinkle score (TWS)”. TWS was calculated as reported elsewhere [31].

Expert evaluation of subjects’ skin smoothness and microrelief was also conducted by a comparison of the Visoface images of the face (frontal, left lateral and right lateral) from T0 and T12 for each subject. The 93 pairs of photographs were assessed using a 3-grade scale (−1: deterioration, 0: no change, +1: improvement). 

### 2.8. Statistical Methods

Statistical analyses were performed using GraphPad Prism (GraphPad Software, version 8.2.0., San Diego, USA) statistical software. For descriptive statistics, Microsoft Excel (Microsoft Corp., version 16.0.12325.20280, Redmond, USA) was used. All the data measured are given as the mean ± standard deviation (SD). The data were tested for normality by a Shapiro–Wilk test. The measured skin parameters were evaluated by descriptive analysis at T0 (baseline) and T12 (after 12 weeks of supplementation). For a comparison of changes between groups at different time points, for scale-level dependent variables a two-way ANOVA with Bonferroni’s multiple comparisons post-hoc test was used. Changes in the TWS scores were statistically evaluated using an independent *t*-test, and for changes in the smoothness and microrelief score, a Wilcoxon rank-sum (Mann–Whitney) test for nonparametric data was used. Values were considered to be statistically significant when *p* was < 0.05.

## 3. Results

**Figure 1:**
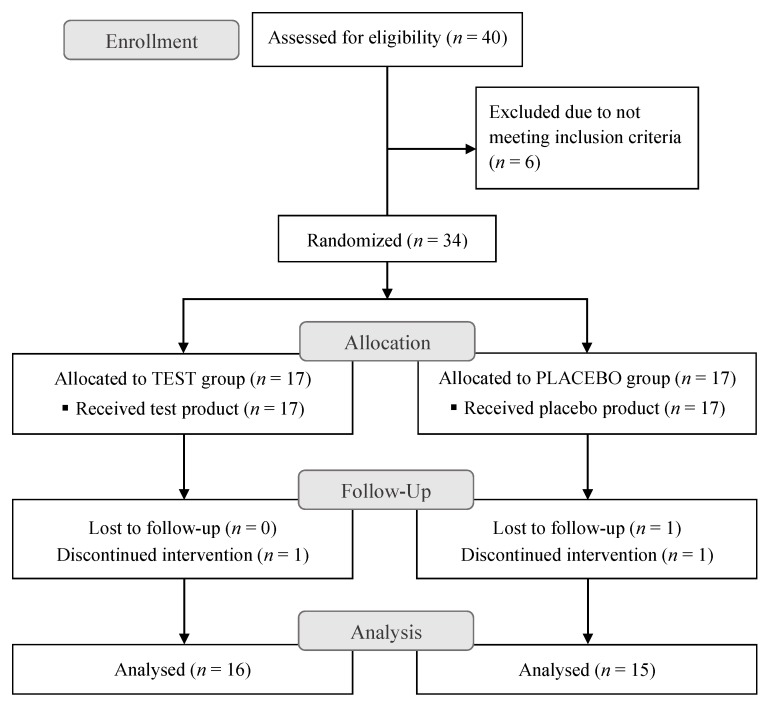
CONSORT (Consolidated Standards of Reporting Trials) flow diagram showing trial design and subjects’ assignment and progression through the trial.

Out of the 34 subjects enrolled in the study, 31 completed the entire 12-week trial (placebo group: 15 subjects, test group: 16 subjects). There were two dropouts in the placebo group and one in the test group, none related to the product intake or the study procedure in general. In both the placebo and test group, one subject discontinued intervention during the trial and in the placebo group one additional subject was lost to follow up at the last regular check. No side effects or adverse events of any kind were reported. The trial design and the flow of subjects through the trial is represented in Figure 1. 

### 3.1. Dermis Density 

**Figure 2:**
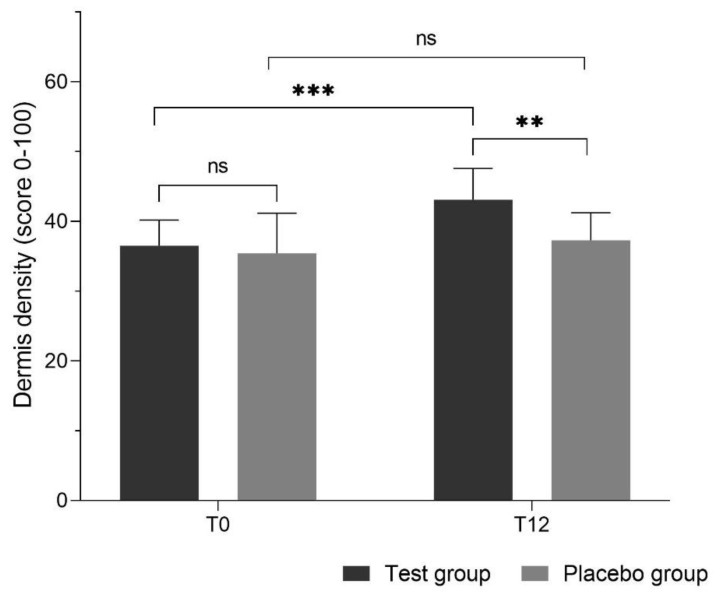
Dermis density before intake (T0) and after 12 weeks of intake of study products (T12). Oral administration of the test product led to increased dermis density after 12 weeks, while in the placebo group there was no significant change over the same period of time. (ns: not significant, ** *p* < 0.01, *** *p* < 0.001).

Figure 2 shows the dermis density before intake of study products (T0) and after 12 weeks of intake (T12). At the beginning of the study, the dermis density between the test and placebo groups was not statistically significantly different (36.5 ± 3.7 vs. 35.4 ± 5.8, *p* > 0.99). After 12 weeks of daily intake of the test products, the density was significantly higher in the test group than in the placebo (43.1 ± 4.5 vs. 37.3 ± 4.0; *p* < 0.005), with a 5.8 increase in comparison to the placebo group (95% CI 1.4, 10.2), corresponding to 16.1% overall increase.

Also, within group comparisons of dermis density show significant increase after 12 weeks of supplementation in comparison to the baseline in the test group (*p* < 0.001), while the change was not significant in the placebo group (*p* > 0.99). 

**Figure 3:**
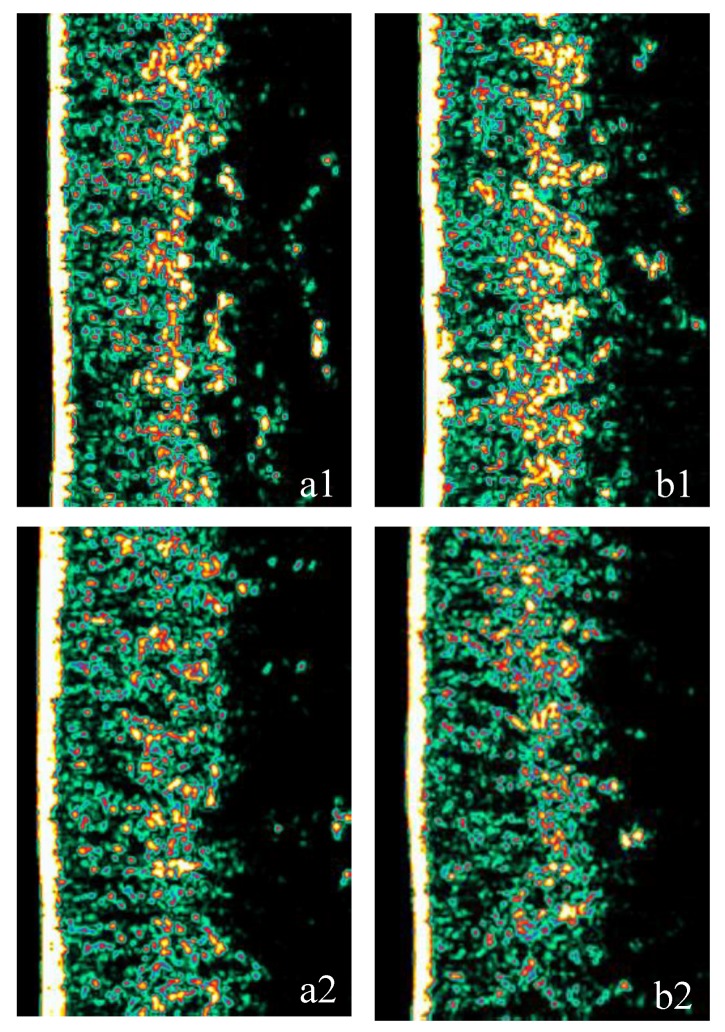
Examples of ultrasound images of the skin before and after 12 weeks of supplementation for a subject from the test group (**a1** and **b1**) and from the placebo group (**a2** and **b2**). In the ultrasound image, the white line to the left represents the epidermis with a water film, to the right followed by dermis, characterized by varying intensities, and subcutis by low-intensity areas due to a homogenous composition. From images a1 and b1, an increase in dermis echogenicity is visible, indicating an improvement in dermal density throughout the study, while slight deterioration can be observed from images (**a2**) and (**b2**). Echogenicity colour scale: white > yellow > red > green > black.

The influence of investigated products on the dermis density can be observed in Figure 3, showing an example of ultrasound images of the skin of two subjects—one from the test group and one from placebo group before the supplementation (Figure 3(a1,a2) respectively) and after 12 weeks of supplementation (Figure 3(b1,b2) respectively). After 12 weeks of supplementation with the test product, echogenicity is visibly increased. 

### 3.2. Dermis Thickness

The starting level of dermis thickness was 1435 ± 110 µm in the test group, and 1564 ± 217 µm in the placebo group, with no statistical difference among them (*p* = 0.21) and after 12 weeks of studying products intake, it remained without significant change in both groups (1469 ± 122 µm and 1591 ± 183 µm, respectively; *p* > 0.99). 

### 3.3. Skin Viscoelasticity

The starting level of viscoelasticity was 1.49 ± 0.38 MPa in the test group, and 1.53 ± 0.64 MPa in the placebo group, with no statistical difference among them (*p* > 0.99). Considering the data relative to the placebo, there was also no significant difference in skin viscoelasticity between the treatment and placebo group after 12 weeks of supplementation (1.38 ± 0.33 MPa and 1.59 ± 0.68 MPa, respectively; *p* > 0.99). 

### 3.4. Skin Hydration

At the beginning of the observation period, no statistically significant difference in the skin hydration between the test and placebo group could be observed (255.5 ± 65.6 µS vs. 230.6 ± 67.3 µS, *p* > 0.99). Considering the data relative to the placebo, there was no significant difference in skin hydration between the treatment and placebo group after 12 weeks of supplementation (200.0 ± 52.3 µS vs. 194.1 ± 44.4 µS, *p* > 0.99). 

### 3.5. Transepidermal Water Loss (TEWL)

At the beginning of the observation period, no statistically significant difference in the TEWL levels between the test and placebo group could be observed. The starting data were 13.2 ± 3.4 g/m^2^/h for the test group and 14.8 ± 5.3 g/m^2^/h for the placebo group (*p* > 0.99). There was no significant difference between both groups after 12 weeks of daily supplementation (13.2 ± 3.4 g/m^2^/h vs. 15.1 ± 5.9 g/m^2^/h, *p* > 0.99). 

### 3.6. Wrinkle Assessments

**Figure 4:**
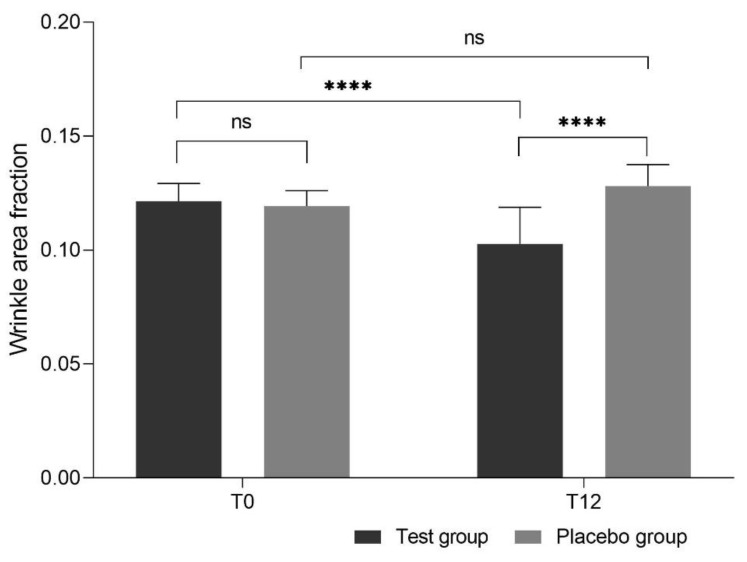
Wrinkle area fraction (mean ± standard deviation (SD)) before intake of study products (T0) and after 12 weeks of intake (T12). Oral administration of test product resulted in decreased wrinkle area in comparison to placebo (ns: not significant, **** *p* < 0.0001).

The effect of the test products on wrinkle expression was assessed in the periorbital area; the results are shown in Figure 4. While measurements of the periorbital wrinkle area fraction showed no significant difference between the test and placebo group initially (0.121 ± 0.008 vs. 0.119 ± 0.007 0.103 ± 0.016, *p* > 0.99), after 12 weeks of supplementation the evaluated wrinkle area fraction was significantly lower in the test group than in the placebo group (0.103 ± 0.016 vs. 0.128 ± 0.009; *p* < 0.0001), with a 0.025 decrease in comparison to the placebo group (95% CI −0.03598, −0.01487), corresponding to a 19.4% overall decrease.

**Figure 5:**
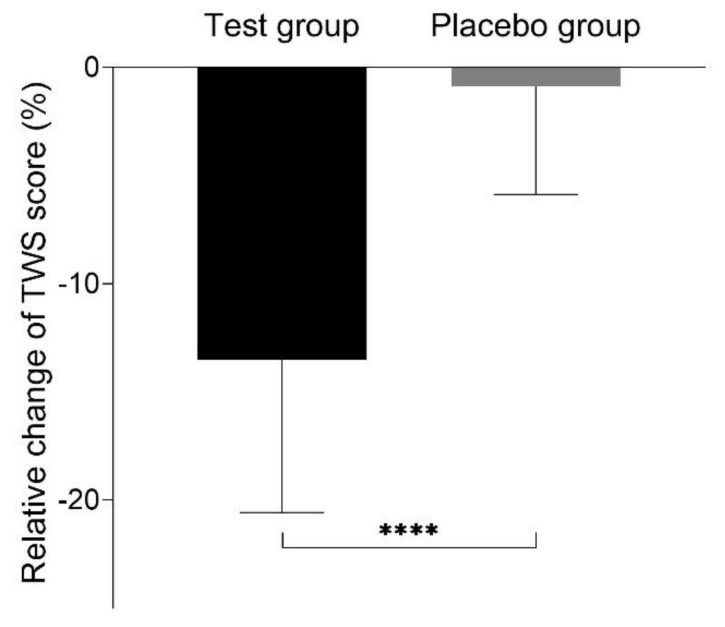
Relative change of total wrinkle score (TWS) from baseline after 12 weeks of supplementation for placebo and test group; **** *p* < 0.0001.

To provide further insight into effects of study products on skin surface, we performed an expert assessment of wrinkles of different types in different face areas (horizontal forehead lines (HF), glabellar frown lines (GF), eye (PO, periorbital lines), nasolabial folds (NL), corner of the mouth lines (CM)) according to the Lemperle scale (0–5) and the total wrinkle score (TWS) was calculated. In the placebo group, no significant change in TWS in comparison to the baseline was observed (9.5 ± 3.2 vs. 9.4 ± 3.1; *p* = 0.39), while in the test group TWS was significantly lower in comparison to the initial values (10.2 ± 4.1 vs. 8.9 ± 3.7; *p* < 0.001). Figure 5 represents the percentage change in TWS from the baseline after 12 weeks of supplementation in both groups.

### 3.7. Skin Smoothness and Microrelief 

The average scores for the expert evaluation of changes in skin smoothness and microrelief lines between baseline and end of supplementation period were determined. While the placebo group had a slight decline in skin smoothness, there was a significant improvement over the study period in the test group (−0.07 vs. 0.44, respectively; *p* < 0.05). 

Although the improvement in the expression of microrelief lines was notably higher in the test group, the change was not statistically significant in comparison to the placebo (0.50 vs. 0.07, *p* = 0.08), which could be also due to the higher variability of scores for microrelief lines.

## 4. Discussion

In the last decade, we have seen an increased use of CoQ10 in health-related products, also those intended to reduce signs of aging. In the market, CoQ10 can be found mostly in food supplements and also as an ingredient in functional foods [32]. For example, it was added as a functional food ingredient to 4% of yoghurts sold in the Slovenian food supply in 2011 [33]. Numerous possible benefits are reported for its role in human health [34,35,36], while it is also an essential component of mitochondrial energy metabolism [37]. In skin, CoQ10 contributes to its outermost barrier against oxidative damage, but its levels in skin as well as other organs decline with age. Due to its perceived ability to protect the skin from free radical damage and reduce signs of aging, Coenzyme Q10 is also used in nutritional supplements supporting skin health, and also cosmetics. *In vitro* experiments show its ability to protect the skin from reactive oxidative species (ROS), reduce damage triggered by UV irradiation, induce the proliferation of skin fibroblasts, inhibit MMPs and accelerate the production of epidermal basement membrane components [38,39,40,41,42,43,44], but properly controlled *in vivo* studies for confirmation of those effects are scarce. The effect of dietary intake of CoQ10 alone (either 50 or 150 mg/day for 12 weeks) on skin parameters was examined in our double-blind, placebo-controlled trial with 33 subjects [14]. Both doses of CoQ10 significantly reduced periorbital wrinkles and microrelief lines and improved skin smoothness. The influence of the CoQ10 dose on the response magnitude was observed only in the expert assessment of wrinkles, as with a higher CoQ10 dose, additional improvements of wrinkles in other facial parts (nasolabial folds, corner of the mouth lines and upper radial lip lines) were observed. However, the CoQ10 supplementation did not significantly affect skin hydration, dermis thickness or density.

In the herein reported study, we investigated the effects of CoQ10 in combination with collagen peptides and vitamins A, C and biotin on the skin parameters. 

Collagen peptides are popular components of nutritional products and supplements for skin health. Besides providing building blocks for collagen (and elastin), it was shown that food-derived collagen peptides stimulate the dermal cellular metabolism, improve the biosynthesis of extracellular matrix proteins and inhibit MMPs [17,44]. Placebo-controlled in vivo studies with collagen as the single active ingredient show positive effects on some skin parameters. In a study with 114 subjects, Proksch et al. showed that the ingestion of 2.5 g of collagen peptides (CP) for 8 weeks increased dermal matrix synthesis, specifically the content of procollagen type I and elastin, and decreased the volume of wrinkles at the outer corner of the eye [15]. In another study with 33 subjects (11/group), it was shown that the ingestion of 2.5 or 5.0 g CP over 8 weeks improved skin elasticity, but there was no significant influence on skin hydration or TEWL [16]. In a study with 33 subjects (11/group), Asserin showed that the ingestion of 10 g of CP (either fish or porcine) over 8 weeks was able to improve skin hydration without influencing TEWL, and, in a study with 106 subjects, that 12 weeks of daily supplementation with 10 g CP improved dermal density [17]. 

The results of the herein reported randomised, double-blind placebo-controlled study of the administration of liquid food supplement, characterised by a combination of water-soluble CoQ10 (50 mg) and fish collagen (4.0 g) over a 12-week period, show several beneficial effects on skin. While in our previous study there were no significant effects on dermal density determined after 12 weeks of supplementation with CoQ10 alone, by the addition of collagen peptides and other nutrients to the test product, significant improvement in dermal density (16.1%) in comparison to the placebo was achieved in the present study, while dermis thickness was not affected. As dermis density is related to the amount of properly structured dermal proteins, e.g., collagen and elastin, the test product may have reduced degradation or promoted the synthesis of structural proteins as shown for collagen peptides in some in vivo [15], animal [45] and in vitro [46] studies as well in some in vitro studies for CoQ10 [39,42,43]. The observed improvement in dermal density is almost double when compared to the previously reported influence of supplementation, with a 2.5-fold dose of CP alone (10 g/day; 8.8% improvement) [17], indicating that some synergy may develop when using CoQ10 and CP simultaneously. The improved structure of the dermis reflected also on the skin surface, as the administration of the test product resulted in significantly decreased measured periorbital wrinkle area and decreased TWS, as well as improved skin smoothness according to expert assessment.

While some previous studies with CP supplementation (10 g/day or 5 g/day) alone showed its ability to improve skin elasticity [16,17,19] and hydration [17,19], others failed to confirm hydration effects [16]. In our present trial, the tested supplement containing CoQ10 and CP did not have any significant effect on elasticity and, similarly to previous reports [16,17], supplementation also didn’t affect TEWL and skin hydration. Although there were no statistically significant changes in skin hydration between the treatment and placebo group after 12 weeks of supplementation, a decreasing trend for hydration was observed in both groups throughout the study period, which can be most likely attributed to seasonal variations. As mentioned, the study was conducted during the autumn and winter months and several studies report dramatic changes in skin surface hydration in this period [47,48].

The strength of this human intervention study was that the effects of the supplementation were evaluated using a series of different skin parameters and compared with a placebo. Some limitations should also be noted. Testing of the supplementation over a longer period would be beneficial, as 12 weeks is quite a short time to detect nutritional effects on skin since the average epidermal skin cycle in healthy skin is 30–40 days [3]. The present study was conducted from late fall to winter, when seasonal drop of skin hydration and elasticity occurs [47,49] due to lower air humidity caused by lower outdoor temperatures and indoor heating. The comparison of the test product to the placebo was therefore essential to overcome such seasonal influences. 

## 5. Conclusions

In the present randomised, double-blind placebo-controlled study, the 12-week administration of the liquid food supplement, characterized by a combination of water-soluble CoQ10 (50 mg) and fish collagen (4.0 g), showed several beneficial effects on skin, as it improved dermis density, reduced measured periorbital wrinkle area as well as TWS and improved skin smoothness. On the other hand, no significant effects of the supplementation on skin hydration, dermis thickness, TEWL and viscoelasticity were determined.
